# Remodelling of myelinated axons and oligodendrocyte differentiation is stimulated by environmental enrichment in the young adult brain

**DOI:** 10.1111/ejn.15840

**Published:** 2022-10-27

**Authors:** Madeline Nicholson, Rhiannon J. Wood, David G. Gonsalvez, Anthony J. Hannan, Jessica L. Fletcher, Junhua Xiao, Simon S. Murray

**Affiliations:** ^1^ Department of Anatomy and Physiology University of Melbourne Parkville Australia; ^2^ Department of Anatomy and Developmental Biology Monash University Clayton Australia; ^3^ Florey Institute of Neuroscience and Mental Health Parkville Australia; ^4^ Menzies Institute of Medical Research University of Tasmania Hobart Australia; ^5^ School of Health Sciences Swinburne University of Technology Hawthorn Victoria Australia; ^6^ School of Allied Health La Trobe University Bundoora Victoria Australia

**Keywords:** activity‐dependent plasticity, differentiation, environmental enrichment, experience‐dependent plasticity, myelin plasticity, myelinated axon, myelination, oligodendrocyte, OPC

## Abstract

Oligodendrocyte production and myelination continues lifelong in the central nervous system (CNS), and all stages of this process can be adaptively regulated by neuronal activity. While artificial exogenous stimulation of neuronal circuits greatly enhances oligodendrocyte progenitor cell (OPC) production and increases myelination during development, the extent to which physiological stimuli replicates this is unclear, particularly in the adult CNS when the rate of new myelin addition slows. Here, we used environmental enrichment (EE) to physiologically stimulate neuronal activity for 6 weeks in 9‐week‐old C57BL/six male and female mice and found no increase in compact myelin in the corpus callosum or somatosensory cortex. Instead, we observed a global increase in callosal axon diameter with thicker myelin sheaths, elongated paranodes and shortened nodes of Ranvier. These findings indicate that EE induced the dynamic structural remodelling of myelinated axons. Additionally, we observed a global increase in the differentiation of OPCs and pre‐myelinating oligodendroglia in the corpus callosum and somatosensory cortex. Our findings of structural remodelling of myelinated axons in response to physiological neural stimuli during young adulthood provide important insights in understanding experience‐dependent myelin plasticity throughout the lifespan and provide a platform to investigate axon–myelin interactions in a physiologically relevant context.

## Graphical abstract

Using environmental enrichment to physiologically induce neuroplasticity in the young adult mouse, we find dynamic structural remodelling of axons and their pre‐existing myelin sheaths. Additionally, we find that oligodendrocytes respond primarily with an increase in differentiation and regionally specific decreases in progenitor density. These results highlight diverse and context‐dependent mechanisms of myelin plasticity and advance our understanding of neurobiology across the lifespan.

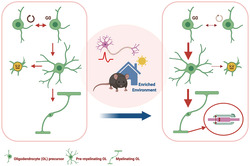

**List of abbreviations**
EdU: 5‐ethynyl‐2′‐deoxyuridine
CNS: central nervous system
EE: environmental enrichment
MBP: myelin basic protein
OPC: oligodendrocyte progenitor cell
P: postnatal day
SEM: standard error of mean
SH: standard‐housed
TEM: transmission electron microscopy

## 1 INTRODUCTION

Oligodendrocytes generate insulating myelin sheaths to facilitate rapid transmission of action potentials along axons in the central nervous system (CNS) and provide essential metabolic and trophic support to neurons (Nave & Werner, 2014). Myelination begins perinatally and peaks at adolescence; however, the production of oligodendrocytes (Dimou et al., 2008; Young et al., 2013) and myelination is ongoing up until at least middle age (Hill et al., 2018; Hughes et al., 2018). This process can be adaptively regulated by neuronal activity (de Faria et al., 2018); however, the underlying cellular mechanisms are incompletely understood. Artificial exogenous stimulation using optogenetic or chemogenetic methods during postnatal development (P19–35) drastically increased oligodendrocyte progenitor cell (OPC) proliferation, subsequently increasing oligodendrocyte differentiation and myelin thickness (Geraghty et al., 2019; Gibson et al., 2014; Mitew et al., 2018). Conversely, non‐invasive suppression of neuronal activity during postnatal development (P21) via social isolation decreased myelin thickness without effecting oligodendrocyte density (Liu et al., 2012; Makinodan et al., 2012) and sensory visual deprivation decreased length of myelin sheaths, but increased oligodendrocyte density (Etxeberria et al., 2016; Osanai et al., 2018). However, when performed in P60 mice pharmacogenetic stimulation resulted in an attenuated increase in OPC proliferation relative to P35 (Mitew et al., 2018), and motor learning paradigms in young adult (P60) rodents (Bacmeister et al., 2020; Keiner et al., 2017; Xiao et al., 2016) exhibited only small increases in new mature oligodendrocyte production. This implies clear experimental paradigm‐ and age‐dependent differences in oligodendroglial and myelin plasticity and warrants a concurrent investigation within a physiologically relevant paradigm, as artificial exogenous stimulation may not accurately reflect native activity states (Venkataramani et al., 2019; Venkatesh et al., 2019).

Environmental enrichment (EE) is a non‐invasive and well‐studied housing paradigm used to induce neuroplasticity with effects that include increased neurogenesis and synaptogenesis, improved learning and memory, and increased white matter volume (Nithianantharajah & Hannan, 2006; Sale et al., 2009). Using EE as a paradigm of physiologically relevant, activity‐dependent plasticity, we investigated the generation of oligodendrocytes and myelin after a 6‐week exposure in 9‐week‐old mice. We found that EE had little effect on gross levels of compact myelin but led to a generalized increase in axon diameter accompanied by thicker myelin sheaths in the corpus callosum. This was associated with paranodal elongation and shortened nodes of Ranvier, together indicative of myelinated axon remodelling. Furthermore, EE uniformly increased the differentiation of oligodendroglia in the corpus callosum and somatosensory cortex but differentially reduced OPC density between region. Collectively, these data identify a new form of myelin plasticity in the dynamic structural remodelling of axons and their pre‐existing myelin sheaths.

## 2 MATERIALS AND METHODS

### 2.1 Experimental animals

C57BL/6 mice were bred and housed in specific pathogen‐free conditions at the Melbourne Brain Centre Animal Facility, in Techniplast IVC cages (Techniplast Group, Italy). All procedures were approved by the Florey Institute for Neuroscience and Mental Health Animal Ethics Committee and followed the Australian Code of Practice for the Care and Use of Animals for Scientific Purposes.

### 2.2 Housing conditions and EdU administration

At 9 weeks of age, male and female animals were randomly assigned to standard or environmentally enriched housing conditions and housed single‐sex with three mice/cage for a period of 6 weeks. Standard‐housed mice remained in shoe‐box sized GM500 cages (Techniplast Group, Italy) with floor area of 501 cm^2^. Enriched mice were moved to GR1800 double‐decker cages (Techniplast Group, Italy) with floor area of 1862 cm^2^ and total height of 38 cm. Enriched cages included a mouse‐house, a selection of rubber dog toys, small plastic objects, tunnels and climbing materials. Standard‐housed mice were provided only basic bedding materials and enriched mice had shredded paper and cardboard. All cages were changed weekly with objects in enriched cages replaced, to maintain object novelty. All mice were given access to food and water ad libitum, and were on a 12‐h light/dark cycle.

Throughout the total housing period of 6 weeks, the drinking water contained thymidine analogue EdU (5‐ethynyl‐2′‐deoxyuridine, Thermo Fisher, cat. no: E10415) to label adult‐born cells. EdU was at a concentration of 0.2 mg/ml, determined previously to be non‐toxic (Young et al., 2013), and refreshed every 2–3 days.

### 2.3 Tissue collection

Mice were anaesthetized and transcardially perfused with 0.1‐M phosphate buffered saline (PBS) followed by 4% EM‐grade paraformaldehyde (PFA, Electron Microscopy Sciences). Brains were dissected and post‐fixed overnight in 4% PFA. The first millimetre of caudal corpus callosum was selected using a coronal mouse brain matrix and micro‐dissected, then placed in Kanovsky's buffer overnight, washed in 0.1‐M sodium cacodylate, and embedded in the sagittal plane in epoxy resin for transmission electron microscopy (TEM) analysis. The remaining brain was rinsed in 0.1‐M PBS, cryoprotected in a 30% sucrose solution, then embedded in OCT and snap‐frozen in isopentane over dry ice, for immunohistochemistry analysis.

### 2.4 Immunohistochemistry and EdU labelling

Twenty‐micrometre coronal cryosections centred around Bregma −1.64 mm were collected in series on SuperFrost plus slides. Sections were incubated overnight at room temperature with primary antibodies diluted in a PBS‐based buffer containing 10% normal donkey serum and 0.3% Triton‐X100, then washed with 0.1‐M PBS, and dark incubated for 4 h at room temperature with appropriate secondary antibodies diluted in the PBS‐based buffer. Thereafter, sections were washed with 0.1‐M PBS, and EdU uptake detected by a 2‐h dark incubation at room temperature with EdU developing cocktail, prepared as per product instructions (Click‐iT™ EdU Alexa Fluor™ 647 Imaging Kit, Thermo Fisher, cat. no: C10340). Sections were washed a final time in 0.1‐M PBS and mounted in DAKO fluorescence mounting medium (Agilent Dako, cat. no: S3023). Primary antibodies included rabbit anti‐Olig2 (Millipore, #AB9610, 1:200), mouse anti‐APC/CC1 (CalBioChem, #OP‐80, 1:200), goat anti‐platelet derived growth factor receptor‐alpha (PDGFRα, R&D systems #AF1062, 1:200), rabbit anti‐contactin associated protein 1 (CASPR, a generous gift from Professor Elior Peles, Weizmann Institute of Science Israel, 1:200) and mouse anti‐voltage gated potassium channel (K_V_1.1, NeuroMab #75‐105, 1:200). Secondary antibodies included donkey anti‐rabbit 594, donkey anti‐mouse 647, and donkey anti‐goat 555 (Alexa Fluor, Invitrogen, 1:200), and donkey anti‐mouse 405 (Abcam, ab175658, 1:200).

### 2.5 Spectral unmixing confocal microscopy imaging and cell counting

Images were obtained using a 20×/0.8NA objective on a Zeiss LSM880 laser scanning confocal microscope with 405‐, 488‐, 561‐, and 633‐nm laser lines and Zen Black 2.3 image acquisition software. Images were taken in Lambda mode, using a ChS1 spectral GaAsP (gallium arsenide phosphide) detector to capture the entire spectrum of light, generating one image containing all fluorophores. An individual spectrum for each fluorophore was obtained by imaging control, single‐stained slides, and these spectra used to segregate each multifluorophore image into a four‐channel image, in a post‐processing step under the linear unmixing function. Uniform settings were used across experiments. Consistent regions of interest (ROI) were maintained across animals; mid‐line corpus callosum and primary somatosensory cortex in the coronal plane at approximately Bregma −1.64 mm, and 3–4 images per ROI per animal were taken.

Images were manually counted by assessors blinded to housing conditions, using ImageJ/FIJI software. Oligodendroglia were defined as Olig2+, OPCs as Olig2+/ PDGFRα+ double‐labelled cells, mature OLs as Olig2+/CC1+ double‐labelled cells and intermediate oligodendroglia as Olig2+/CC1−/PDGFRα−. The area of corpus callosum and somatosensory cortex was measured in each image and counts expressed as cells/mm^2^.

### 2.6 Spectral confocal reflectance (SCoRe) microscopy imaging and analysis

Twenty‐micrometre thick coronal sections were imaged on a Zeiss LSM880 laser scanning confocal microscope with a 40×/1.0 NA water immersion objective using 488‐, 561‐, and 633‐nm laser lines passed through the Acousto‐Optical Tunable Filters 488–640 filter/splitter and a 20/80 partially reflective mirror. Compact myelin reflected light that was then collected using three photodetectors set to collect narrow bands of light around the laser wavelengths (Gonsalvez, Yoo, et al., 2019; Hill et al., 2018; Schain et al., 2014). Uniform settings were used across experiments. Images were acquired in tile scans of 5‐μm z‐stacks, at a minimum z‐depth of 5 μm from the surface. ROIs were consistent with spectral images and three to four images per ROI per animal were taken. Image analysis was performed in ImageJ/FIJI. Maximum intensity z‐projection images were applied a minimum threshold cut‐off and the resulting area of positive pixels in each ROI measured.

### 2.7 Confocal microscopy imaging and analysis for paranodes and nodes of Ranvier

Images of immunostained sections were obtained using a 40×/1.3 NA oil objective on a Zeiss LSM880 laser scanning confocal microscope using 561‐ and 633‐nm laser lines and Zen Black 2.3 image acquisition software. Images were taken as 2 × 1 tile scans (10% overlap) in z‐stacks of approximately 5 μm. A consistent region of interest of coronal, midline corpus callosum centred around Bregma −1.64 mm was maintained across sections. Three images per slide were taken.

For each image, 40–50 pairs of paranodes with clear adjacent K_V_1.1 juxtaparanodes and an interspaced node of Ranvier were manually identified and measured using the line tool in Image J/FIJI software. Paranode density was assessed as individual count per 100 μm^2^, using an automated macro. Briefly, images were thresholded using “moments,” noise removed using “despeckle,” a total count generated using “analyse particles” excluding edges and anything <50 pixels^2^, and ROI area measured.

### 2.8 Transmission electron microscopy and analysis

Semi‐thin (0.5–1 μm) sections of caudal corpus callosum centred around Bregma −2.30 mm were collected on glass slides in the sagittal plane and stained with 1% toluidine blue, for ROI identification. Subsequent ultrathin (0.1 μm) sections were collected on 3 × 3 mm copper grids and contrasted with heavy metals. Ultrathin sections were viewed using a JEOL JEM‐1400Flash TEM. Images were captured using the JEOL integrated software and a high‐sensitivity sCMOS camera (JEOL Matataki Flash). Eight‐ten distinct fields of view were imaged at 10,000× magnification per animal. FIJI/Image J image analysis software (National Institutes of Health) was used to count myelinated axons and the Trainable WEKA Segmentation plugin (Leslie & Heese, 2017) used to segregate myelin, allowing use of the magic wand tool to measure inner and outer axon areas, to obtain axon diameter distribution and to calculate inner and outer diameters for g‐ratio calculation. For each animal, at least 100 axons were measured. Resin embedding, sectioning, post‐staining and EM imaging was performed at the Peter MacCallum Centre for Advanced Histology and Microscopy.

### 2.9 Statistical analysis

All data were analysed and graphed in GraphPad Prism vs8 software. Sample size was determined based on what is generally used in the field. EM data was analysed using a two‐way analysis of variance (ANOVA) with Sidak's multiple comparison test or unpaired two‐tailed *t* test. Spectral and SCoRe imaging data were analysed using an unpaired two‐tailed *t* test. Data are presented as mean ± SEM. A significance threshold *p* value of 0.05 was used.

## 3 RESULTS

### 3.1 EE does not alter gross levels of compact myelin but promotes myelinated axon remodelling

To physiologically induce neuronal activity, we adopted the well‐established EE housing paradigm (Nithianantharajah & Hannan, 2006). Nine‐week‐old mice were housed under EE or standard‐housed (SH) control conditions for a period of 6 weeks and the thymidine analogue EdU was administered continuously in the drinking water to label adult‐born cells. At 15 weeks of age, mice were perfused and brain tissue collected. The EE housing paradigm is well established to induce hippocampal neurogenesis (Nithianantharajah & Hannan, 2006). To verify that our EE housing paradigm exerted this effect, we analysed the density of EdU+ cells in the dentate gyrus and observed a significant increase in EE mice compared with SH control mice (862 ± 108.7 cells/mm^2^ compared with 453 ± 10.1 cells/mm^2^, *p* = 0.02, Student's *t* test, *n* = 3–4 mice/group, data = mean ± SEM). This indicated that our paradigm was inducing neuronal plasticity as expected.

**FIGURE 1:**
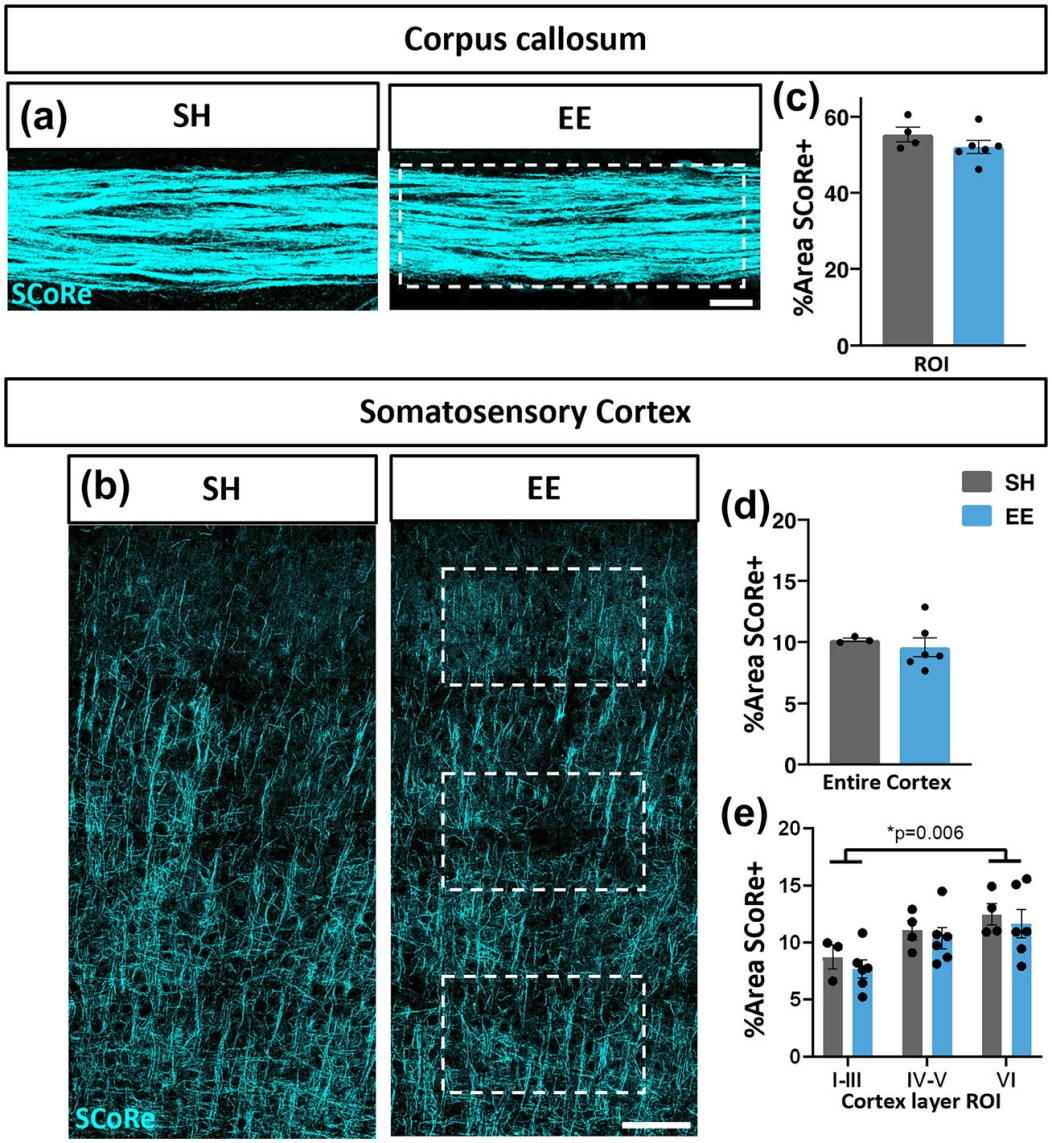
Environmental enrichment does not alter the percentage area of compact myelin detected by SCoRe. (a,b) Representative confocal images of SCoRe+ signal for control SH mice and EE housed mice from coronal sections of the mid‐line corpus callosum (a) and somatosensory cortex (b). (c–e) Quantification of the percentage area positive for SCoRe signal measured in the corpus callosum ROI (*p* = 0.25, unpaired two‐tailed *t* test) (c), across the entire somatosensory cortex (*p* = 0.62, unpaired two‐tailed *t* test) (d) and in three layer‐specific cortical ROIs (dotted outlines, interaction: *p* = 0.99; factors: layer *p* = 0.006, housing *p* = 0.33, two‐way ANOVA with Sidak's multiple comparisons) (e). c–e: *n* = 3–6 mice/group, data = mean ± SEM. Scale bar = a: 50 μm, b: 100 μm

We next assessed the effect of EE on myelin plasticity by using SCoRe microscopy to detect gross levels of compact myelin in the corpus callosum (Figure 1a), as representative of a key white matter tract, and in the overlying region of somatosensory cortex (Figure 1b) from which callosal fibres originate, as substantial cortical myelin acquisition occurs during young adulthood (Hill et al., 2018; Hughes et al., 2018). There was no change in the overall percentage area of compact myelin between EE and SH control mice in the corpus callosum (Figure 1c), indicating that EE during young adulthood induced no significant increase in the gross level of myelination. Similarly, in the somatosensory cortex, we observed no significant change in the percentage area of compact myelin following EE housing (Figure 1d). Additional layer‐specific analysis revealed that deeper cortical layers were more heavily myelinated than superficial layers (Figure 1e, factor: layer *p* = 0.006, housing *p* = 0.33), confirming that SCoRe microscopy has the capacity to detect 5–10% differences in level of myelination; however, no layer‐dependent difference was found between housing conditions. These data suggest there is no significant increase in gross level of myelination following EE during young adulthood in either corpus callosum or somatosensory cortex.

**FIGURE 2:**
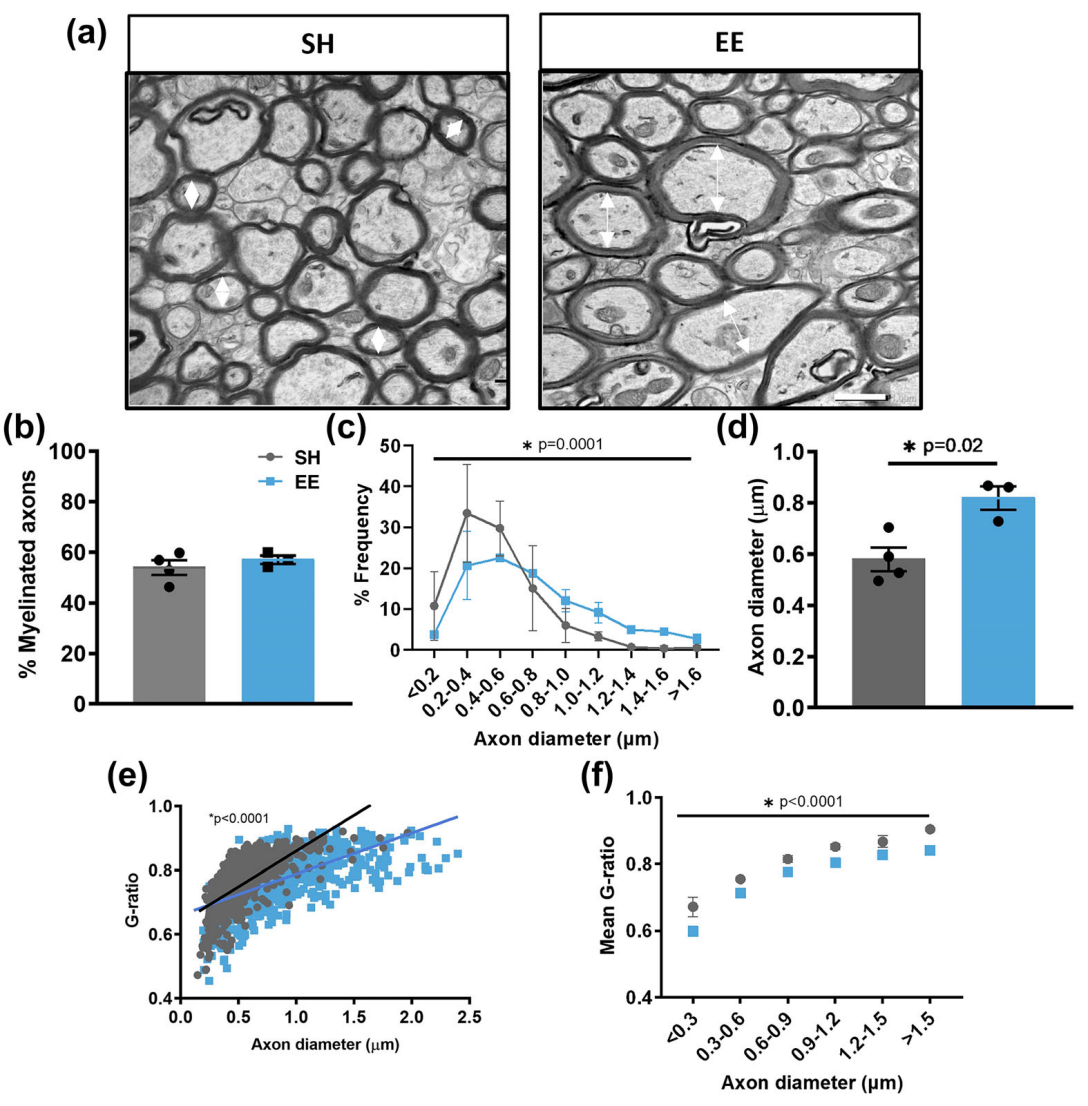
Environmental enrichment increases both axonal calibre and pre‐existing myelin sheath thickness in the corpus callosum. (a) Representative EM micrographs of corpus callosum of control SH or EE housed mice. Small arrows indicate diameters <1 μm (SH) or >1 μm (EE). Scale bar = 1 μm. (b) Quantification of the percentage of myelinated axons in the corpus callosum (*p* = 0.45, unpaired two‐tailed *t* test). (c) Percentage frequency distribution of myelinated axon diameter (Chi^2^ test; *p* = 0.0001). (d) Quantification of mean axon diameter in the corpus callosum (*p* = 0.02, unpaired two‐tailed *t* test). (e) Scatter plot of individual G‐ratio relative to axon diameter (*p* < 0.0001, linear regression analysis). (f) Plot of average g‐ratio binned by axon diameter (two‐way ANOVA with Sidak's multiple comparison test; factors: housing *p* < 0.0001, diameter *p* < 0.0001, interaction *p* = 0.8). For c–f: >100 axons/mouse/group, *n* = 3–4 mice/group, data = mean ± SEM

After establishing that EE does not induce gross changes in compact myelin levels, we sought next to assess whether EE had an effect on the structural parameters of myelinated axons and we examined the corpus callosum using transmission electron microscopy (TEM; Figure 2a). Concordant with the SCoRe imaging data, we found no significant change in the percentage of myelinated axons between housing conditions (Figure 2b), confirming that EE had no effect on de novo myelination of previously unmyelinated axons in the corpus callosum. Surprisingly, there was a shift to the right in the frequency distribution of myelinated axon diameter in EE mice compared with SH control mice (Figure 2c, *p* = 0.0001), with a reduction in the proportion of myelinated axons with small diameters (0.2–0.6 μm) and a corresponding increase in the proportion of myelinated axons with large diameters (>1 μm). This suggests that EE resulted in a general increase in the diameter of myelinated axons. Indeed, on average there was a ~ 30% increase in myelinated axon diameter in EE mice compared with SH control mice (Figure 2d, *p* = 0.02). Intriguingly, this coincided with a reduction in G‐ratio of myelinated axons in EE mice compared with SH controls (Figure 2e, *p* < 0.0001), indicative of thicker myelin sheaths. The mean G‐ratio was decreased uniformly across all myelinated axon diameters (Figure 2f, *p* < 0.0001), indicating a generalized effect of EE in promoting thicker myelin. In line with axon and myelin growth, we also observed an increase in the vertical height of the corpus callosum in the coronal plane following EE (216.0 ± 6.07 μm compared with 143.2 ± 1.30 μm *p* < 0.0001, Student's *t* test, *n* = 3–4 mice/group, data = mean ± SEM), suggesting enlargement of corpus callosal volume. Together, these results strongly suggest that EE in young adult mice has no overt influence on the generation of de novo myelin sheaths. Instead, EE appears to induce the combined dynamic remodelling of both the axon and its myelin, whereby axon diameter is increased and myelin sheath growth is reinitiated.

**FIGURE 3:**
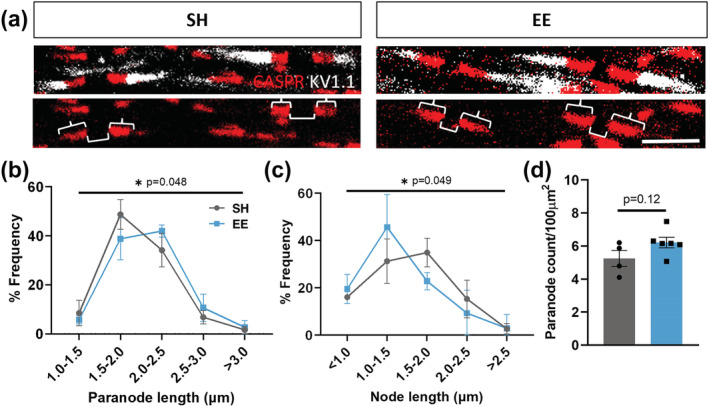
Environmental enrichment results in the lengthening of paranodes and shortening of nodes of Ranvier in the corpus callosum. (a) Representative confocal micrographs of paranode (CASPR, brace) pairs with interspersed node of Ranvier (bracket), demarcated by juxtaparanodes (K_V_1.1). (b) Percentage frequency distribution of paranode length (μm) (Chi^2^ test; *p* = 0.048). (c) Percentage frequency distribution of node of Ranvier length (μm) (Chi^2^ test; *p* = 0.049). (d) Quantification of paranode density as per total paranode count/100 μm^2^ (*p* = 0.12, unpaired two‐tailed *t* test). For b,c: 120–150 pairs/mouse/group, *n* = 4–6 mice/group, data = mean ± SEM

We reasoned that a reinitiation of myelin sheath growth to increase sheath thickness would result in an elongated internode and therefore increased paranode length. To further investigate the effect of EE on myelinated axon remodelling, we used immunohistochemistry to assess paranodes and adjacent nodes of Ranvier within the corpus callosum (Figure 3a). We measured 120–150 pairs of CASPR+ paranodes with interspaced nodes of Ranvier per mouse and observed a global shift to the right in the distribution of paranode lengths in EE mice compared with SH control mice (Figure 3b, *p* = 0.048), indicating paranodal lengthening. Concurrently, we observed a shift to the left in the distribution of node of Ranvier lengths of EE mice compared with SH control mice (Figure 3c, *p* = 0.049), indicating nodal shortening. As this analysis was restricted to paranode–node pairs, we counted the number of paranodes within the corpus callosum and observed no difference in paranode density between housing groups (Figure 3d, *p* = 0.12). This result further confirms no significant effect of EE in enhancing the de novo generation of myelin sheaths. Together, our results provide clear evidence that young adult CNS myelination is dynamically adaptive to activity‐dependent plasticity induced by EE. Importantly, we show this response does not involve an overt generation of de novo myelin sheaths on previously unmyelinated axon segments, but instead a remodelling of existing myelin sheaths associated with changes in calibre of the underlying axon.

### 3.2 EE during young adulthood induces oligodendrocyte differentiation globally, and has regionally distinct effects on OPC homeostasis

**FIGURE 4:**
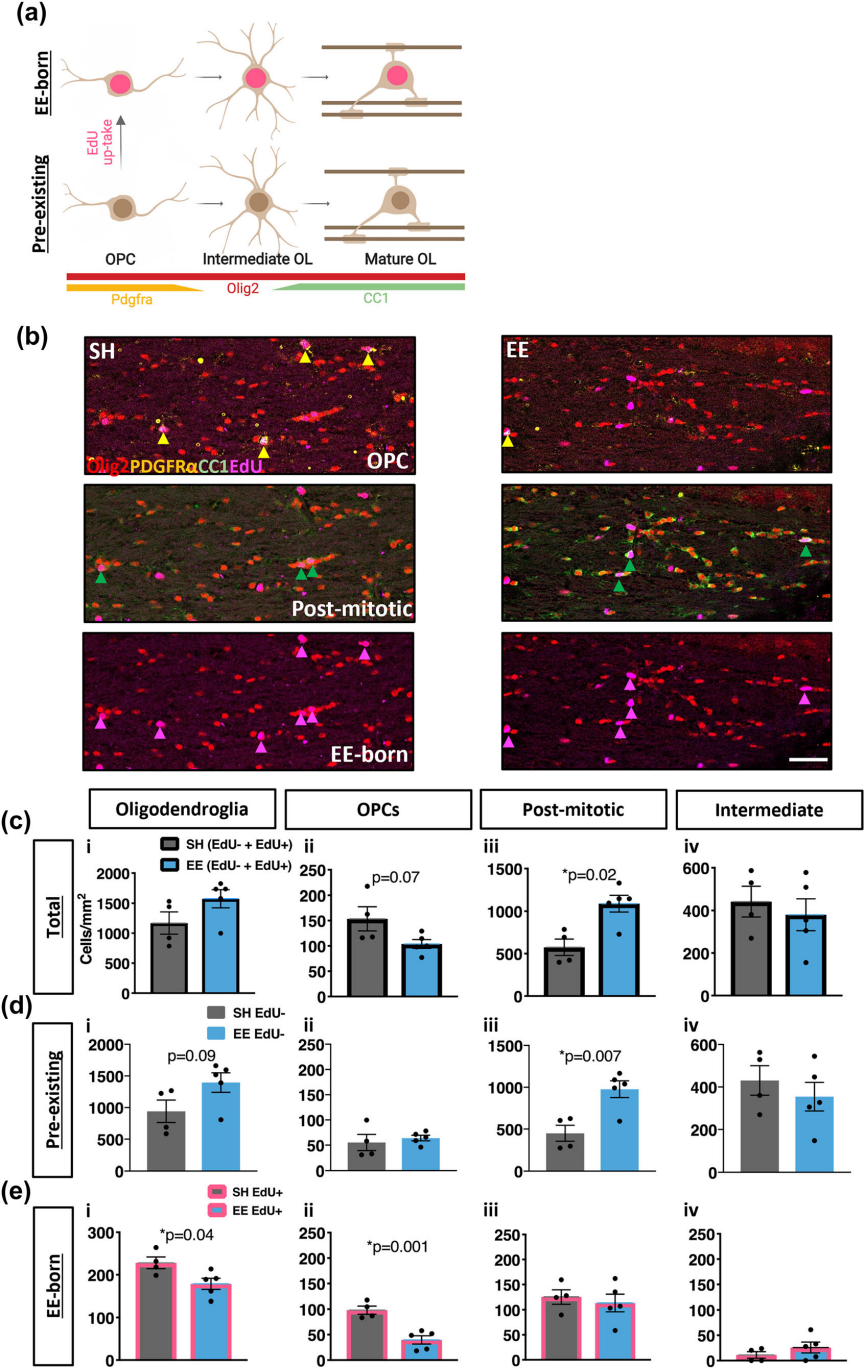
Environmental enrichment induces oligodendrocyte differentiation and reduces OPC proliferation in the corpus callosum. (a) Schematic of immunolabels to distinguish oligodendroglial lineage: Olig2 to identify all oligodendroglia, CC1 to identify post‐mitotic oligodendrocytes, PDGFRα to identify OPCs and CC1‐/PDGFRα‐ to identify intermediate oligodendroglia. EdU reactivity detects oligodendroglia born during 6 weeks of environmental enrichment. This image was created using BioRender.com. (b) Representative confocal micrographs of quadruple‐immunostained corpus callosum for EE and SH housed mice. Yellow arrows indicate EdU+Olig2+Pdgfrα+ OPCs, green arrows indicate EdU+Olig2+CC1+ post‐mitotic oligodendrocytes, and pink arrows indicate EdU+ EE‐born oligodendroglia. (c–e) Quantification of oligodendroglial densities in the corpus callosum: (i) total oligodendroglia (EdU− and EdU+; oligodendrocytess *p* = 0.13, (ii) OPCs *p* = 0.07, (iii) post‐mitotic oligodendrocytes *p* = 0.02, (iv) intermediate *p* = 0.58) (c), pre‐existing oligodendroglia (EdU−; (i) oligodendroglia *p* = 0.09, (ii) OPCs *p* = 0.58, (iii) post‐mitotic oligodendrocytes *p* = 0.007, (iv) intermediate *p* = 0.46) (d) and EE‐born oligodendroglia (EdU+; (i) oligodendoglia *p* = 0.04, (ii) OPCs *p* = 0.001, (iii) post‐mitotic oligodendrocytes *p* = 0.62, (iv) intermediate *p* = 0.21) (e). c–e: *n* = 4–5 mice/group, unpaired two‐tailed *t* test, data = mean ± SEM

As we had detected changes in myelin sheath remodelling in the corpus callosum, we next examined whether EE also influenced oligodendrocyte generation and differentiation in this region. To examine this, we co‐immunolabelled sections of corpus callosum with Olig2 (to identify all cells in the oligodendroglial lineage), CC1 (to identify post‐mitotic mature oligodendrocytes) and PDGFRα (to identify OPCs) (Figure 4a,b). There was a small population of cells that were Olig2+/CC1‐/PDGFRα‐ that we considered to be intermediate oligodendroglia (Figure 4a).

After 6 weeks of EE, there was no change in the density of total oligodendroglia (Figure 4c(i)). Breaking this population down, we observed a trend decrease in OPCs (Figure 4c(ii), *p* = 0.07), but a significant increase in the density of mature oligodendrocytes of EE mice compared with SH mice (Figure 4c(iii), *p* = 0.02). There was no change in the density of intermediate oligodendrocytes (Figure 4c(iv)).

As the drinking water contained the thymidine analogue EdU throughout the 6‐week EE housing period, this enabled us to further examine the dynamics of oligodendrocyte generation and differentiation in the corpus callosum. Oligodendroglia pre‐existing before the EE Intervention would be EdU−, and oligodendroglia generated during the EE period (EE‐born) would be EdU+ (Figure 4a). There was a trend increase in the density of pre‐existing oligodendroglia following EE (Figure 4d(i), *p* = 0.09). This was almost exclusively due to a significant increase in mature oligodendrocytes (Figure 4d(iii), *p* = 0.007), as no change was detected in the density of either OPCs (Figure 4d(ii)) or intermediate cells (Figure 4d(iv)). This suggests that EE caused an increase in the direct differentiation and/or survival of pre‐existing oligodendroglia.

We then assessed oligodendroglia that were born during EE (EdU+) and surprisingly this revealed a decrease in the density of total EE‐born oligodendroglia (Figure 4e(i), *p* = 0.04). This was almost entirely due to a decrease in the density of EE‐born OPCs (Figure 4e(ii), *p* = 0.001), as the density of mature (Figure 4e(iii)) and intermediate (Figure 4e(iv)) oligodendrocytes was similar to the SH mice. This is indicative of reduced OPC proliferation during the enrichment period, possibly due to enhanced direct differentiation. Overall, these data indicate that EE during young adulthood promotes maturation and/or survival of pre‐existing oligodendroglia in the corpus callosum, which impairs OPC turnover.

**FIGURE 5:**
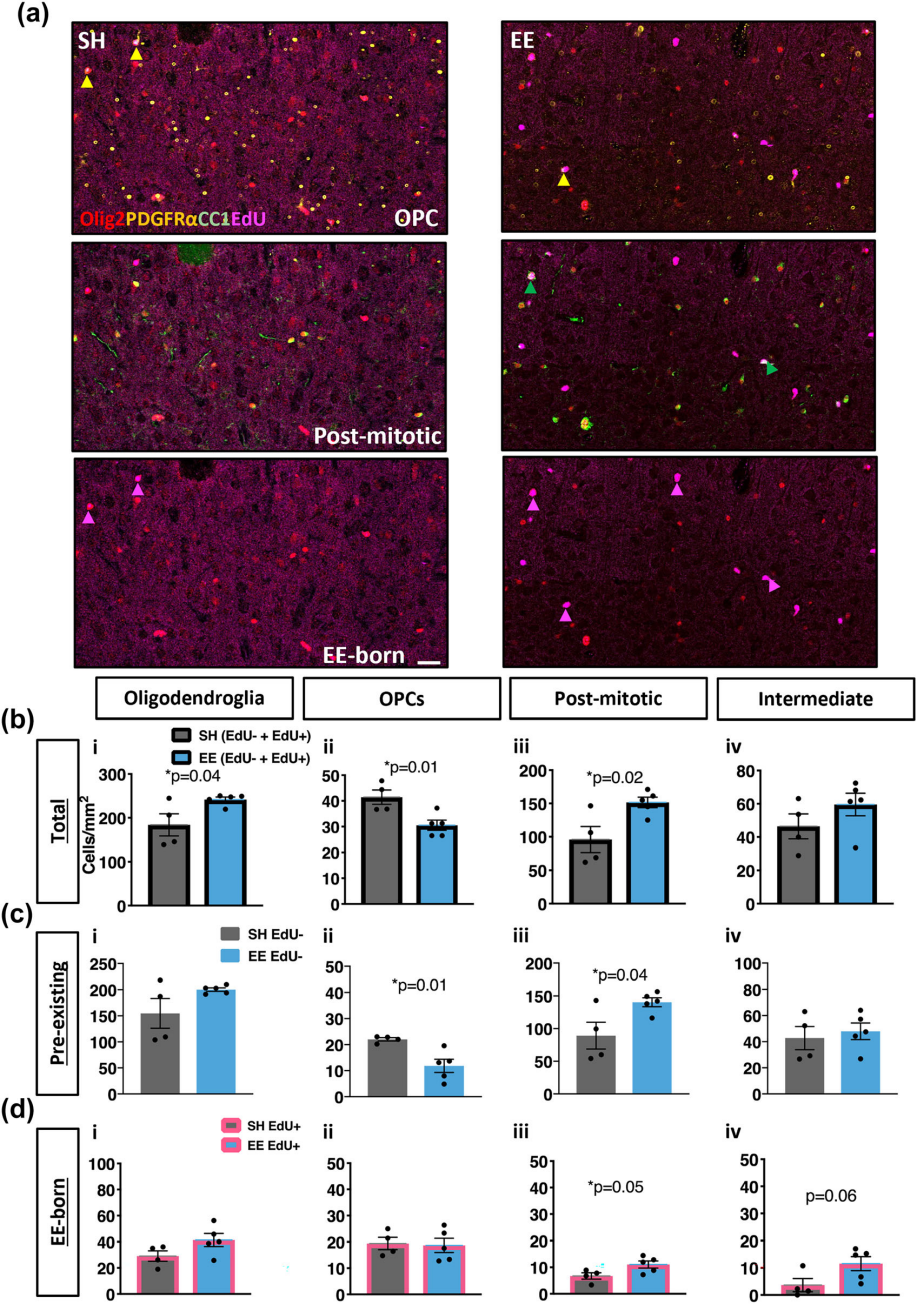
Environmental enrichment induces oligodendrocyte differentiation in the somatosensory cortex. (a) Representative confocal micrographs of quadruple‐immunostained somatosensory cortex for SH and EE house mice. Yellow arrows indicate EdU+Olig2+Pdgfrα+ OPCs, green arrows indicate EdU+Olig2+CC1+ post‐mitotic oligodendrocytes (none in SH image), and pink arrows indicate EdU+ EE‐born oligodendroglia. (b–d) Quantification of oligodendroglial densities in the somatosensory cortex: Total oligodendroglia ((i) oligodendroglia *p* = 0.04, (ii) OPCs *p* = 0.01, (iii) post‐mitotic oligodendrocytes *p* = 0.02, (iv) intermediate *p* = 0.24) (b), pre‐existing oligodendroglia ((i) oligodendroglia *p* = 0.11, (ii) OPCs *p* = 0.01, (iii) post‐mitotic oligodendrocytes *p* = 0.04, (iv) intermediate *p* = 0.64) (c), and EE‐born oligodendroglia ((i) oligodendroglia *p* = 0.11, (ii) OPCs *p* = 0.85, (iii) post‐mitotic oligodendrocytes *p* = 0.05, (iv) intermediate *p* = 0.06) (d). b–d: *n* = 4–5 mice/group, unpaired two‐tailed *t* test, data = mean ± SEM

Considering we observed marked changes in oligodendrocyte generation in the corpus callosum, we also assessed oligodendroglia in the somatosensory cortex to examine whether EE was exerting similar or disparate influences in this region (Figure 5a). Unlike in the corpus callosum, in the somatosensory cortex, 6 weeks of EE induced an increase in the density of total oligodendroglia (Figure 5b(i), *p* = 0.04). Similar to the corpus callosum, we observed a decrease in the density of OPCs (Figure 5b(ii), *p* = 0.01) and an increase in the density of mature oligodendrocytes of EE mice compared with SH controls (Figure 5b(iii), *p* = 0.02). There was no change in the density of intermediate oligodendrocytes (Figure 5b(iv)).

We then examined the pre‐existing oligodendroglia specifically and observed an unchanged total density (Figure 5c(i)), but in contrast to the corpus callosum, there was a reduction in the density of pre‐existing OPCs of EE mice compared with SH controls (Figure 5c(ii), *p* = 0.01). Similar to the corpus callosum, there was an increase in the density of pre‐existing post‐mitotic oligodendrocytes in EE mice compared with SH controls (Figure 5c(iii), *p* = 0.04) and unchanged density of intermediate oligodendroglia (Figure 5c(iv)). This suggests that EE uniformly promotes the direct differentiation and/or survival of pre‐existing oligodendroglia, but differentially influences OPCs.

To further explore the influence of EE on oligodendrocyte generation in the somatosensory cortex, we then examined the EE‐born (EdU+) population of oligodendroglia specifically. In contrast to the corpus callosum, we saw no change in the density of total EE‐born oligodendroglia (Figure 5d(i)) or EE‐born OPCs (Figure 5d(ii)). Instead, we observed a small increase in the density of EE‐born post‐mitotic mature oligodendrocytes in EE mice compared with SH controls (Figure 5d(iii), *p* = 0.05) and a trend increase in the density of EE‐born intermediate oligodendroglia (Figure 5d(iv), *p* = 0.06). These data suggest that in the somatosensory cortex of young adult mice, EE does not affect recruitment of OPCs to the cell cycle but rather accelerates the differentiation of both pre‐existing and newly generated oligodendroglia.

In summary, EE in young adult mice, which is representative of a physiological level of neuronal stimulation, primarily promotes the differentiation of pre‐existing oligodendroglia in both the corpus callosum and somatosensory cortex. However, in the corpus callosum, this results in a reduced density of newly generated OPCs, whereas in the somatosensory cortex, the density of pre‐existing OPCs is reduced.

## 4 DISCUSSION

In this study, we demonstrate that physiological stimulation induced by EE in the young adult CNS promotes the dynamic remodelling of myelinated axons. We observed no effect of EE on gross levels of compact myelin coverage in the corpus callosum or somatosensory cortex; however, we identified the dynamic growth of axon calibre in the corpus callosum along with increased myelin thickness. This resulted in elongated paranodal lengths and shortened nodes of Ranvier. Additionally, EE specifically promoted the differentiation of OPCs and pre‐myelinating oligodendrocytes in both the corpus callosum and somatosensory cortex, which resulted in regionally distinct alterations to OPC density. Together, these findings present dynamic remodelling of the existing axon–myelin unit as an important component of physiological, activity‐dependent plasticity and raise important mechanistic questions for future investigation.

### 4.1 Physiological plasticity involves dynamic remodelling of the myelinated axon unit in the young adult CNS

We demonstrated that EE exposure in 9‐week‐old mice promoted axon calibre enlargement in the corpus callosum. The mid‐sagittal area of corpus callosum was shown to increase following 2 months of EE in 4‐month‐old rats (Markham et al., 2009) and decrease in 18‐month‐old rhesus monkeys raised from 2 to 12 months in social isolation (M. M. Sánchez et al., 1998), indicating the housing environment can have a sustained and bidirectional effect on callosal growth, and with our results suggest this is predominantly due to fluctuations in axon diameter. Consistent with this, 4 months of EE in 14‐ to 24‐month‐old rats resulted in increased diameter predominantly of smaller diameter axons (<1 μm) in the corpus callosum (Yang et al., 2013) and blocking auditory input in 6‐week‐old mice reduced the average diameter predominantly of the smallest (<3.5 μm) axons in the trapezoid body of the brainstem (Sinclair et al., 2017). Collectively, this suggests that smaller diameter axons are most plastic. Rapid dynamic increases in axon calibre have been reported in hippocampal slices following high frequency stimulation that enlarged synaptic boutons upon a time scale of 10–30 min, and correspondingly increased axon diameters by ~5% (Chéreau et al., 2017). Although perhaps mechanistically different, our data confirm physiological relevance of structural plasticity and suggest that over an extended period of stimulation, the overall axon diameter can continue to increase up to 30%. Specialized actomyosin networks enable temporary axonal radial contractility to allow axon transport of cargo larger than the actual diameter (Wang et al., 2020), suggesting there are structural molecular substrates that could theoretically reorganize to mediate long‐term change. There has been limited study of activity‐dependent axonal structural changes (Costa et al., 2018; Lazari et al., 2018) and our results of long‐term, physiologically induced axon calibre growth in vivo establish EE as a paradigm for future investigation of this phenomena.

Interestingly, alongside axon calibre growth, we observed an increased thickness in overlying myelin sheaths, suggesting EE induced a reinitiation of myelin growth (Fields & Dutta, 2019; Snaidero & Simons, 2017; Zuchero et al., 2015). Molecularly, myelin sheath thickness could be increased by the sustained activation of extracellular signal‐regulated kinases 1 and 2 (Erk1/2) in mature oligodendrocytes which promoted the excess synthesis of myelin proteins and lipids (Jeffries et al., 2016), or through hyperpolarization‐activated, cyclic nucleotide‐gated (HCN2) channels that regulate resting membrane potential in myelinating oligodendrocytes to determine myelin sheath length, by aligning local translation of MBP protein with activity levels (Swire et al., 2021). However, it is unclear whether the current increase in myelin thickness was independently initiated or driven secondarily by the increase in axon diameter. Artificially increasing axon calibre by deleting neuronal *Pten* in cerebellar granule cells to activate the AKT1‐mTOR pathway was sufficient to induce the myelination of these normally unmyelinated axons (Goebbels et al., 2016), suggesting axon growth precedes myelin thickness changes. However, the expansion in axon calibre may be reliant on myelin as during retinal ganglion development oligodendrocyte‐derived signals triggered the local neurofilament accumulation and network reorganization that enabled axon enlargement (I. Sánchez et al., 1996) and unhealthy myelin induced by sulphated loss reduced axon calibre (Marcus et al., 2006). Mechanistically, myelin may support the maintenance of axon diameter through secreted extracellular vesicles that enhance axonal transport to increase stress tolerance and firing rate (Fröhlich & Kuo, 2014), as loss of the myelin proteins PLP and CNP leads to deficiencies in axonal transport and secondary axonal degeneration (Frühbeis et al., 2020). It is also possible that axon–myelin changes were induced concurrently, as onset of developmental myelination induced the secretion of axonal vesicles that formed a feedback loop to instruct sheath elongation and stabilization (Almeida et al., 2021). Future investigations utilizing inducible genetic models to fluorescently label new myelin would be important in enabling precise distinction between the dynamics of axon–myelin responses below new and existing myelin sheaths. Importantly, the current results are a clear demonstration of axon–myelin unit remodelling in a non‐invasive, physiologically induced paradigm and determining the underlying intercellular mechanisms will be critical.

This dynamic myelinated‐axon remodelling resulted in elongated paranodes and shortened nodes of Ranvier. These components are important in determining the speed of conduction of an action potential along an axon (Arancibia‐Cárcamo et al., 2017; Ford et al., 2015), suggesting our results represent functional changes required in adapting to a new environment. Dynamic axon–myelin sheath interactions to decrease periaxonal space and lengthen node of Ranvier resulted in quickened conduction velocity and correlated with improved spatial Radial Arm Maze learning (Cullen et al., 2021). Conversely, enlarged periaxonal space and shortened node length slowed conduction timing (Cullen et al., 2021), to facilitate fine motor skill learning (Tang et al., 2018). Impaired forelimb motor learning due to desynchronized spike‐time arrivals through long‐range axons has been associated with genetic myelination deficits (Kato et al., 2019), and blocking oligodendrogenesis impaired the coupling of hippocampal sharp wave ripples and cortical spindles required for contextual fear learning (Steadman et al., 2019). Together, these studies suggest that dynamic interactions between myelin internodes and their axons are necessary for cognitive plasticity by functioning to regulate conduction timing and coordinate neuronal activity.

### 4.2 Physiological plasticity primarily promotes oligodendroglial differentiation in the young adult CNS

We demonstrated that EE in 9‐week‐old animals predominantly induced oligodendroglial differentiation without prior proliferation, in both corpus callosum and somatosensory cortex. Increased survival of newly generated oligodendroglia in the amygdala of 8‐week‐old mice following EE has been observed (Okuda et al., 2009) and the terminal differentiation and integration of cortical OPCs in 8‐ to 14‐month‐old mice was increased fivefold with sensory stimulation, up from 22% during basal conditions (Hughes et al., 2018). Similarly, increased oligodendroglial survival and differentiation occurred after 2 weeks of repetitive transcranial magnetic stimulation in 12‐week‐old mice, without changing the density of EdU+ OPCs (Cullen et al., 2019). These results in adult animals stand in contrast to invasive optogenetic (Geraghty et al., 2019; Gibson et al., 2014) and pharmacogenetic (Mitew et al., 2018) paradigms in juvenile (P19–35) animals that induced drastic increases in OPC proliferation, prior to differentiation, suggesting subtle, but critical paradigm‐ and age‐related differences in oligodendrocyte plasticity. In fact, the current EE‐induced increase in oligodendrocyte differentiation resulted in impaired OPC homeostasis with a global decrease in OPC density. However, there was regional heterogeneity with decreased density of EE‐born, divided OPCs in the corpus callosum and decreased pre‐existing, non‐divided OPCs in the somatosensory cortex. Homeostatic asymmetric OPC differentiation is rare (Hughes et al., 2013) and can be influenced by intrinsic or environmental factors (Boda et al., 2015), suggesting the current results represent regional heterogeneity in the proliferation of replacement OPCs, which is potentially accounted for by spatiotemporal variation in the ratio of ion channels expressed by OPCs known to influence cell cycle entry (Spitzer et al., 2019). Cell cycle length appears extremely consistent across the lifespan with the fraction of cells dividing defining the production rate (Gonsalvez, Craig, et al., 2019), and there is a drastic spatiotemporal decline in OPC density during development (P9–60) (Gonsalvez, Craig, et al., 2019; Nicholson et al., 2018). Perhaps it is simply the cell intrinsic constrains of cell cycle length and total number that limit OPC responsivity to neuronal activity in adulthood. Young adult mice (P60) (Mckenzie et al., 2014; Mitew et al., 2018) do exhibit attenuated increases in newly generated OPCs in response to neuronal stimulation as compared with juvenile mice (P19–35) (Gibson et al., 2014; Mitew et al., 2018). Furthermore, it could be that a prolonged enhancement of differentiation in adulthood eventually begins to outpace replacement and slowly depletes the OPC pool, which would indicate a physiological maximum to enhancing oligodendrogenesis in adulthood. This would have implications for designing remyelinating or other oligodendroglial‐targeted therapies for adult‐based brain injury or disease contexts. Indeed, carefully timed motor learning paradigms have proven efficacious in improving recovery from a demyelinating injury, but only when onset of training is slightly delayed (Bacmeister et al., 2020).

In this current study, we observed no significant increase in gross levels of compact myelin coverage following EE, which appears contradictory to the concurrent increase in oligodendrocyte density observed. However, it takes up to 28 days (Cullen et al., 2019) for a new oligodendrocyte to generate a full complement of myelin sheaths and perhaps in the current paradigm increased myelination would be observed at a time point 4 weeks post‐EE. Further, periaxonal space and nodal remodelling occurred in mice unable to produce new oligodendrocytes (Cullen et al., 2021) and prolonged EE exposure throughout development (P15–90) led to thicker myelin in absence of greater oligodendrocyte density (Goldstein et al., 2021). Together, these three findings are key in demonstrating that myelinated axon remodelling occurs within mature oligodendrocytes independent of de novo generation of myelin sheaths. In summary, these current results contribute to a growing acknowledgement that physiological plasticity in adult animals is mediated by relatively small increases in oligodendroglial integration and myelinated axon remodelling.

## CONFLICT OF INTEREST

The authors declare no conflicting financial or other interests.

## AUTHOR CONTRIBUTION

Madeline Nicholson: Conceptualization, data curation, formal analysis, funding acquisition, investigation, methodology, project administration, visualization, writing – original draft, writing – review & editing. Rhiannon Wood: Investigation, methodology, project administration, writing – review & editing. David Gonsalvez: Methodology, writing – review & editing. Anthony Hannan: Conceptualization, methodology, funding acquisition, writing – review & editing. Jessica Fletcher: Methodology, project administration, supervision, writing – review & editing. Junhua Xiao: Conceptualization, methodology, project administration, resources, funding acquisition, supervision, writing – review & editing. Simon Murray: Conceptualization, funding acquisition, project administration, resources, supervision, writing – review & editing.

### PEER REVIEW

The peer review history for this article is available at https://publons.com/publon/10.1111/ejn.15840.

## Data Availability

Data will be made freely available upon direct request to the authors'.
